# Identification of a novel familial FGF16 mutation in two cases of MF4

**DOI:** 10.1186/1753-6561-9-S1-A19

**Published:** 2015-01-14

**Authors:** B Jones, H Byers, S Watson, WG Newman

**Affiliations:** 1 Manchester Centre for Genomic Medicine, The University of Manchester, Oxford Rd, Manchester M13 9PL, UK

## Background

Metacarpal 4-5 fusion (MF4) is a rare congenital hand malformation characterised primarily by ulnar deviation of the fifth finger, clinodactyly, shortening of the fifth metacarpal and reduced mobility of the fifth finger. A small number of familial cases have been described in the literature, consistent with X-linked recessive inheritance. In May 2013 causative mutations in the FGF16 gene were identified in two unrelated patients with MF4.[1] This prompted the sequencing of FGF16 in half-brothers with MF4, with a view to identifying a possibly causative mutation.

## Methods

DNA samples from the phenotypically unaffected mother and her two affected sons were amplified using PCR and then underwent dye terminator chemistry based Sanger sequencing of the FGF16 gene, using primers for all three of its exons and their flanking intronic regions.

## Results

In all three individuals sequenced, a novel frameshifting 19 base duplication (c.275_293dup) was identified in exon 2 of FGF16, for which the mother was heterozygous, and both her affected sons were hemizygous. This mutation is predicted to lead to the introduction of a premature stop codon and therefore a loss of function of the affected allele. The predicted protein sequence change is p.(Ser98Argfs*28).

## Conclusions

In the context of the identification of mutations in FGF16 in other MF4 patients,[1] there is strong evidence that the duplication in exon 2 of FGF16 identified in this family is causative for the diagnosis of MF4 in these two males. The mother is heterozygous for a mutation in this gene, and consistent with reports of other female carriers is unaffected. Additionally, identification of Fgf16 expression in interdigital spaces in a mouse embryo indicates its involvement in hand patterning, again suggesting that this mutation is significant.

